# Hybrid Swarm Intelligence and Human-Inspired Optimization for Urban Drone Path Planning

**DOI:** 10.3390/biomimetics10030180

**Published:** 2025-03-14

**Authors:** Yidao Ji, Qiqi Liu, Cheng Zhou, Zhiji Han, Wei Wu

**Affiliations:** 1 School of Mechanical Engineering, University of Science and Technology Beijing, Beijing 100083, China; yidaoji@ustb.edu.cn (Y.J.); c.zhouustb@gmail.com (C.Z.); 2 Institute of Unmanned Systems, Beihang University, Beijing 100191, China; liuqiqi422@gmail.com; 3 College of Engineering, Ocean University of China, Qingdao 266404, China; hanzhiji@ouc.edu.cn; 4 State Key Laboratory of Multimodal Artificial Intelligence Systems, Institute of Automation, Chinese Academy of Sciences, Beijing 100190, China

**Keywords:** urban drone, path planning, swarm intelligence algorithm, human-inspired algorithm

## Abstract

Urban drone applications require efficient path planning to ensure safe and optimal navigation through complex environments. Drawing inspiration from the collective intelligence of animal groups and electoral processes in human societies, this study integrates hierarchical structures and group interaction behaviors into the standard Particle Swarm Optimization algorithm. Specifically, competitive and supportive behaviors are mathematically modeled to enhance particle learning strategies and improve global search capabilities in the mid-optimization phase. To mitigate the risk of convergence to local optima in later stages, a mutation mechanism is introduced to enhance population diversity and overall accuracy. To address the challenges of urban drone path planning, this paper proposes an innovative method that combines a path segmentation and prioritized update algorithm with a cubic B-spline curve algorithm. This method enhances both path optimality and smoothness, ensuring safe and efficient navigation in complex urban settings. Comparative simulations demonstrate the effectiveness of the proposed approach, yielding smoother trajectories and improved real-time performance. Additionally, the method significantly reduces energy consumption and operation time. Overall, this research advances drone path planning technology and broadens its applicability in diverse urban environments.

## 1. Introduction

Over the past decade, drones have increasingly become a frequent presence in news reports and everyday activities. Public perception of drones has shifted from novelty to normalcy. Particularly in terms of civil applications, drones have been commonly seen in activities ranging from celebratory performances to security monitoring, from power line inspections to package delivery. In other words, the effective use of drones not only significantly facilitates daily life but has also become one of the key pillars supporting the low-altitude economy [1,2,3,4]. Consequently, the capability to command drones to perform tasks with stability, efficiency, and economy has become a key technological objective for both academia and industry. Among the core technologies is path planning for drones in three-dimensional space [5,6,7], which addresses the challenge of generating a reasonable path for drones to avoid obstacles and reach predetermined destinations in complex, dynamic environments. This is a practical yet challenging task that has attracted numerous researchers to explore and optimize drone paths using optimization algorithms.

Zhou et al. tackled the issue of drone trajectory replanning by employing a gradient-based algorithm to optimize newly generated paths. They also proposed a path-guided optimization approach, which combines efficient sampling-based topological path searching and parallel trajectory optimization to address issues of local minima and path optimality [8]. Melo et al. investigated dynamic full-coverage path planning for drones by combining linear and heuristic optimization algorithms. They further estimated energy costs at different flight speeds and utilized fog-edge computing to reduce computational overheads [9]. Liu et al. developed a graph-based point-to-point path-planning algorithm that first generates two routes using the elliptic tangent graph method, then applies four heuristic rules to select an efficient, collision-free path [10]. Peng et al. addressed the planning of multiple waypoints along a path, formulating the objective function to focus on energy-efficient offloading and safe path planning [11]. From the perspective of unknown environments, Venkatasivarambabu et al. proposed a Dynamic Window approach that integrates Dynamic Programming and Probabilistic Route Mapping techniques to enhance drone navigation and localization capabilities [12]. Han et al. focused on optimizing the mapping process. They developed a modeling method based on geographical coordinates subdividing grids and combined A* and backtracking path-planning algorithms to reduce the computational complexity of indoor drone path planning while improving planning reliability [13]. Chen et al. proposed a bilevel optimizer to address the rapid trajectory planning of drones under constraints of variable time allocation and safety sets. The optimizer solved a low-level convex quadratic program and updated the high-level spatial and temporal waypoints [14]. Souto et al. explored optimization strategies using popular artificial intelligence techniques, employing simple Q-learning, ε-greedy methods, and a state–action–reward–state–action framework to optimize paths while considering urban terrain, weather, and drone energy consumption [15].

It is worth noting that optimization algorithms based on mathematical and computational theories have advantages in terms of quick convergence and low computational complexity when solving drone path-planning problems. However, for more complex and robustness-demanding path optimization problems, bio-inspired optimization algorithms have gained greater attention. For example, Shivgan and Dong formulated the drone path-planning problem as a traveling salesman problem and used genetic algorithms to find optimal routes that minimize energy consumption [16]. Yuan et al. also applied genetic algorithms to generate and optimize full-coverage scanning paths for drones. They utilized the Good Point Set Algorithm to generate initial populations and designed heuristic crossover operators and random interval inverse mutation operators to avoid local optima, ultimately achieving better flight efficiency [17]. Mesquita and Gaspar optimized drone patrol paths using Particle Swarm Optimization (PSO) to better monitor and deter birds, focusing on maximizing the random generation of paths and waypoints [18]. Huang, inspired by the dynamic divide-and-conquer strategy and A* algorithm, improved the PSO algorithm by dividing complex planning problems into small-scale subproblems with fewer waypoints. The uniformity of particle expansion was evaluated, ultimately generating paths for drones in three-dimensional space [19]. Phung and Ha proposed an improved PSO method based on spherical vectors, describing paths between waypoints with vectors and considering constraints such as path length, threats, turning angles, and flight altitude to generate safety-enhanced flight paths [20]. Ying et al. proposed to introduce a Bayesian model on the basis of the genetic algorithm, considering the rules and general habits of seafarers, and showed that the sea collision avoidance route generated by this algorithm is more effective than that of the pure genetic algorithm [21]. Cao et al. introduced a path replanning algorithm based on threat assessment using a dynamic Bayesian network, which ensures that unmanned underwater vehicles can adjust their paths to avoid danger when facing uncertain events [22].

Similar to PSO, Ant Colony Optimization (ACO) is another widely utilized optimization algorithm. Inspired by the foraging behavior of ants, ACO mimics these natural phenomena to solve optimization problems. Wan et al. modeled the three-dimensional path planning problem as a multi-objective, multi-constraint optimization problem. By improving modeling and search capabilities, the proposed ACO algorithm maintained both global and local search abilities and achieved a balanced and diverse Pareto solution set [23]. In [24], ACO was used to address path generation in complex environments, where specific workloads, capacity constraints, and mobility speeds were considered for each node. Comparative studies demonstrated the efficiency and feasibility of the improved ACO algorithm over conventional methods. The social structures and behaviors of certain species often inspire new optimization algorithm designs. For instance, Zhang et al. focused on the Grey Wolf Optimization Algorithm (GWOA), based on hierarchical predatory behavior. They introduced dynamic adjustment strategies for nonlinear convergence and weight coefficients, verifying the algorithm’s effectiveness in planning drone paths in complex environments [25]. Yu et al. combined the Differential Evolution algorithm with GWOA to enhance the exploration capability of path-planning algorithms. They modified GWO’s search strategies and adjusted DE’s mutation strategy based on ranking concepts [26]. Similarly, Jiang et al. proposed a dual-layer planning strategy that utilized a collision-avoidance speed controller based on a partially observable Markov decision process and improved GWOA using an enhanced communication mechanism and ε-level comparison for path planning [27].

Pan et al. improved the golden eagle optimizer by integrating personal example learning and mirror reflection learning strategies into the algorithm framework, effectively enhancing the efficiency of drone routes for power inspections [28]. Zhang et al. focused on Harris Hawks Optimization, introducing Cauchy mutation strategies, adaptive weights, and the Sine–Cosine Algorithm to improve algorithm performance [29]. Shen et al. adopted strategies such as beta distribution, Levy distribution, and two different cross-operators to enhance the dung beetle optimizer. Interestingly, the behavioral patterns of slime molds also serve as references for optimization algorithms [30]. Abdel-Basset et al. used Pareto optimality to balance multiple objective functions in drone path planning, thereby improving the performance of a hybridized slime mold algorithm [31]. The relationship between behaviors can also provide optimization pathways. For example, Zu et al. drew inspiration from the hunter–prey optimization algorithm to enhance the rapid path-planning capabilities of drones. They designed a chaotic mapping model and a golden sine strategy to improve algorithm updates and convergence speed [32]. Zhang et al. improved the search and rescue optimization algorithm, which has the advantage of being easy to apply but suffers from slow convergence in drone path planning. Their main improvements included integrating a heuristic crossover strategy and a real-time path adjustment strategy [33].

Drawing inspiration from biological behaviors and strategies enhances drone path-planning algorithms, improving their ability to handle multi-objective and multi-constraint scenarios while mimicking biological intelligence. The main contributions of this study are summarized as follows:

- Inspired by the collective intelligence of animal groups and electoral process in human societies, this study introduces hierarchical structures and group interaction behaviors into the standard PSO algorithm. Specifically, competitive and supportive behaviors are mathematically modeled, significantly enhancing the learning strategies of particles and improving the algorithm’s global search capability during its mid-term optimization stage.
- To prevent the algorithm from falling into local optima during the later stages of optimization, a mutation mechanism is introduced. This enhancement further improves the diversity of the population, thereby increasing the overall accuracy of the improved PSO algorithm.
- To address the challenges in drone path planning, this paper proposes an innovative method that integrates a path segmentation and prioritized update algorithm with a cubic B-spline curve algorithm. These methods effectively improve the optimality and smoothness of the generated paths, ensuring safe navigation for drones in complex urban environments. Additionally, the proposed approach outperforms other swarm optimization algorithms in terms of path length.

The remainder of this paper is organized as follows: Section 2 introduces the standard PSO algorithm; details of our algorithm enhancements are presented in Section 3; simulation and analysis of the improved algorithm are discussed in Section 4; the application of the improved algorithm in drone urban path planning is presented in Section 5; and Section 6 summarizes the research and future directions of this paper.

## 2. The Standard Particle Swarm Optimization (PSO) Algorithm

PSO is an optimization algorithm based on swarm intelligence, inspired by the behaviors of bird flocks searching for food in the environment. The core concept of the algorithm involves modeling the positions of food searched for by the bird flock as the potential solutions to problem. Then, each individual in the bird flock is abstracted as a particle with no mass or volume. At last, the continuous movement of particles, driven by interactions, is treated as the process of searching for the optimal solution in a multi-dimensional solution space. Due to its simplicity, efficiency, and ease of implementation, the PSO algorithm has gained widespread attention for solving various optimization problems [34].

In the standard PSO algorithm, each particle represents a potential solution to the optimization task. During each iteration, each particle adjusts its position based on not only the best position which has been discovered (pbest) but also the best position which has been found by the entire swarm (gbest). Through this mechanism, the PSO algorithm continuously explores the solution space and optimizes until the optimal solution is found. Specifically, the basic workflow of the standard PSO algorithm is as follows. Firstly, set various parameters of the PSO algorithm. Secondly, randomly initialize the position and velocity of each particle in the swarm. Thirdly, calculate the fitness value for each particle, representing the quality of the solution it finds, and update the pbest and gbest if the new fitness value is better than the pbest or gbest. Then, update the velocity and position of each particle using specific update equations with pbest and gbest. At last, repeat the steps of calculating and updating until a predefined stopping criterion is met, such as reaching the maximum number of iterations or achieving a solution quality in a specified threshold.

In the above algorithmic process, the particle update phase is the most critical step. Mathematically, this can be described as follows. For a D-dimensional optimization problem, each particle has two numerical characteristics: the velocity vector and the position vector, represented as $V_{i}=[v_{i1},v_{i2},\cdots ,v_{iD}]$ and $X_{i}=[x_{i1},x_{i2},\cdots ,x_{iD}]$, respectively, where $V_{i}$ and $X_{i}$ denote the velocity vector and the position vector of the $i_{th}$ particle, respectively. $v_{ij}$ and $x_{ij}$ denote the velocity and position of the *j*-th dimension of the $i_{th}$ particle, respectively. During the search process in the solution space, each particle updates its velocity and position according to the following equations:

(1) $$\left\{\begin{array}{l} v_{ij}^{t+1}=\omega v_{ij}^{t}+c_{1}r_{1}(pbest_{ij}^{t}-x_{ij}^{t})+c_{2}r_{2}(gbest_{j}^{t}-x_{ij}^{t})\\ x_{ij}^{t+1}=x_{ij}^{t}+v_{ij}^{t+1} \end{array}\right.$$

where t denotes the current iteration number. $v_{ij}^{t}$ and $x_{ij}^{t}$ denote the velocity and position of the $j_{th}$ dimension of the $i_{th}$ particle during the $t_{th}$ iteration, respectively. ω denotes the inertia weight of the particle. $c_{1}$ and $c_{2}$ denote the cognitive and social learning factors, respectively, both positive real numbers. $r_{1}$ and $r_{2}$ are random numbers uniformly distributed in the range of [0, 1]. $pbest_{ij}^{t}$ denotes the *j*-th component of the pbest of the $i_{th}$ particle during the $t_{th}$ iteration. $gbest_{j}^{t}$ denotes the *j*-th component of the gbest during the $t_{th}$ iteration.

## 3. Improved Particle Swarm Optimization Algorithm

### 3.1. Electoral Process

#### 3.1.1. Introduction of Electoral Process

According to numerous studies, the topology of the population in swarm intelligence algorithms, that is, the connection structure between all individuals in the whole population, determines the mode of information exchange in the swarm, which indirectly affects the performance of the algorithm, such as convergence speed and accuracy. At the same time, the learning strategy of individuals in swarm intelligence algorithms, that is, the way in which individuals update their position or speed according to interactive information, determines the individual’s exploration (finding new solutions) and exploitation (improving current solutions) behaviors in the search space, which directly affects the performance of the algorithms. Therefore, the standard PSO algorithm may have the following shortcomings: Firstly, the individual in the standard PSO algorithm relies too much on the current global optimal information, which may lead to the algorithm converging to the current global optimal solution prematurely, that is, the local optimal solution in the solution space. Secondly, since each particle interacts with all particles in the standard PSO algorithm, i.e., the fully connected topology, this may lead to a rapid reduction in the diversity of solutions (particles) in the optimization process, that is, rapid homogenization, which affects the convergence accuracy of the algorithm. Last, the topology of the standard PSO algorithm is static and cannot be adaptively adjusted according to the optimization situation, so the search ability of the algorithm may decrease with the optimization process. And for the learning strategies, the standard PSO algorithm adopts a monotonous formula to update the velocities and positions of particles, which may lead to a lack of effective balance between exploration and exploitation behavior. The fixed learning strategy of the standard PSO algorithm will also lead to a gradual decrease in the search efficiency of the algorithm.

To address the limitations of the standard PSO algorithm and expand its applications in fields such as path planning, this study proposes an improved version of the PSO algorithm by introducing an electoral process. This mechanism draws inspiration from human electoral processes and the social interaction behaviors, including periodically electing, leading, competing, and supporting. By refining the algorithm’s topological structure and learning strategies, these behaviors are simplified and incorporated into the algorithmic framework. The aim is to enhance the algorithm’s search capability and accuracy. The specific improvements are detailed below.

#### 3.1.2. Hierarchical Structure

**Figure 1:**
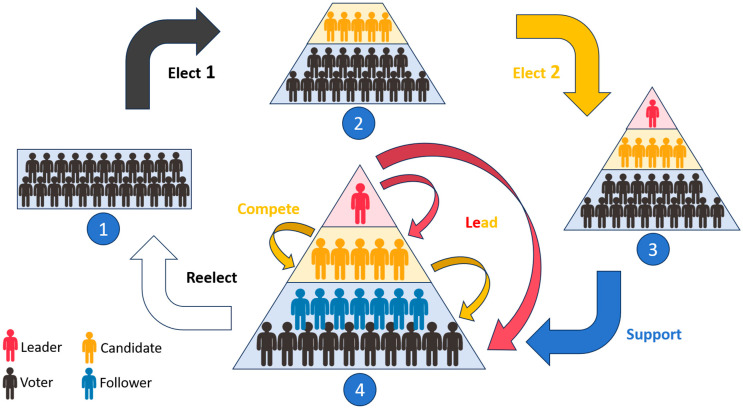
A graphic description of hierarchy and behaviors in PSO.

To emulate the social interactions observed in human electoral processes, this study introduces a hierarchical structure to classify the particle population within the Improved Particle Swarm Optimization (IPSO) algorithm. Based on their fitness values, the particles are divided into four tiers: leader, candidates, voters, and followers. The hierarchical structure is illustrated in Figure 1.

In order to emulate the social interaction behaviors observed in human electoral processes, this study first introduces a hierarchical structure to divide the particles within the IPSO algorithm, that is, the particles in the IPSO algorithm are divided into four levels according to the fitness value: leader, candidates, voters, and followers. The specific structure is shown in Figure 1. The leader is the particle with the lowest fitness value in the population (for the minimization problem), which is represented by the red man in Figure 1, and the candidates are the N particles in the population with the fitness value second only to the leader, which are represented by the orange men in Figure 1, while the rest of the particles are voters and followers represented by the black and blue men in Figure 1, which need to be divided according to the support rate.

The leader is the particle with the lowest fitness value in the population and represents the global optimal solution found so far. The leader provides a clear search target for the entire population, guiding all particles closer to the optimal solution, which is the key to algorithm convergence, similar to the current global optimal experience (gbest) in the standard PSO algorithm.

The candidates are the particle swarm second only to the leader in fitness value, representing multiple local optimal solutions. They represent the good individuals in the population, which are the objects of supporting voters and potential candidates to become the leader. The presence of candidates allows for the preservation of the suboptimal solutions that have been found to maintain population diversity, while increasing the exploitation of suboptimal solutions, thereby improving search efficiency.

The voters are the largest particle swarm in the population, and they are the most important population in the IPSO algorithm, which conducts extensive exploration with the guidance of the leader and candidates in the search space to find possible optimization solutions. What’s more, these particles support the candidates in the upper level according to the change in their fitness values, and affect the search direction of the entire population by adjusting the support rate for each candidate. The diversity of voter particles and exploratory behavior are the keys for the algorithm to avoid precocious convergence and maintain search breadth.

The voters are the largest particle swarm in the population, and they are the most important in the IPSO algorithm. Guided by leaders and candidates, they explore extensively in the search space for possible optimization solutions. In addition, these particles will support the candidate particles in the upper level according to the change in their fitness values, and then adjust their search direction according to the different support objects and support rates, thus affecting the search direction of the entire population. The diversity of the voters and their variable search behaviors are the key for the IPSO algorithm to avoid precocious convergence and maintain the search breadth.

The followers are a subgroup of the voters whose support rates for the candidates exceed a predefined threshold. They have absolute loyalty to the candidates they follow and no longer support other candidates. The presence of followers reinforces the focused search for good candidates (exploitation).

#### 3.1.3. Interaction Behaviors in Electoral Process

The human electoral processes include many complex processes, such as periodic elections, open campaigns, voting support, and so on. And these processes include a variety of human interaction behaviors. In order to facilitate the introduction of the electoral process, this study simplifies and summarizes the various human interaction behaviors in the electoral processes into periodically electing, leading, competing, and supporting. These behaviors are shown in Figure 1.

*The behavior of periodically electing*. This behavior refers to the process of re-electing the leader and candidates according to a fixed time period. It is a key process to ensure that the leader and candidates always represent the global optimal solution and the suboptimal solutions, so as to ensure that the search direction of the entire population can be updated in time. The arrows Reelect, Elect1, and Elect2 are used in Figure 1 to represent this behavior. Specifically, the Elect1 process first sorts the voters and followers by their fitness values. Then, it selects the top N better particles to compare with the current candidates. If the fitness value of any voter is better than any candidate, this voter will be promoted to the new candidate. At the same time, it resets the support rate of the replaced candidates’ followers to the initial support rate, that is, the followers of the replaced candidates become voters. Subsequently, the Elect2 process selects the optimal particle as the new leader in the updated candidates, thereby guiding the entire population to evolve towards the optimal solution. The Reelect process is to periodically trigger calls to Elect1 and Elect2 to update the leader and candidates. This dynamic electoral process not only gives the population an updated global optimal search direction but also enhances the diversity of the population, which helps the algorithm to jump out of the local optimal and improve the search ability of the global optimal solution.

*The behaviors of leading and competing*. In the PSO algorithm introduced into the electoral process in this paper, these two behaviors are the key interaction behaviors. The behavior of leading is reflected in the way the voters follow the candidates based on their fitness values, as well as how all particles follow the leader, which is represented by three Lead arrows in Figure 1. Specifically, the orange Lead arrow indicates that in each iteration, the voters follow the candidates based on the support rates, that is, the candidates lead the voters. The two red Leader arrows indicate that the leader leads the candidates, voters, and followers. This behavior of leading provides a clear search direction for the population. And the behavior of competing is embodied in the candidates, which decide whether to introduce the competition term through a certain probability called the competition rate, represented by the Compete arrow in Figure 1. The competition term is designed to be the average position of the other candidates, similar to the center of mass, in order to make each candidate move away from other candidates, which is the key to simulate the individual differences and dynamic changes in social competition. This behavior of competing can push candidates away from the average position of other candidates, increasing the likelihood of exploring new areas, thereby enhancing the diversity of the population. Through this combination of the behaviors of leading and competing, the algorithm can effectively simulate the social hierarchical structure and competition mechanism, thereby enhancing the search ability and adaptability of the algorithm.

*The behavior of supporting*. This behavior is one of the cores of dynamic population interaction, and it is also a key basis for the dynamic conversion of voters and followers. Supportive behavior refers to adjusting one’s search direction based on the degree of support from voters and followers for the candidates, which is represented by the Support arrow in Figure 1. This degree of support is quantified in this paper through the indicator of the support rate. And the voters and followers will adjust their support rate for the candidates according to the changes in their own fitness values after each iteration. For voters, this behavior enables the voters to respond more flexibly to dynamic changes within the population, allowing the better candidates to attract more voters, thereby facilitating a concentrated search and accelerating the convergence speed. At the same time, this can also increase the diversity of the population, as different voters may support different candidates, resulting in a fragmentation of search directions and reducing the risk of the algorithm falling into local optimums. For followers, this behavior strengthens the focused search for good candidates, allowing the algorithm to explore more deeply in the identified promising areas, which can improve the efficiency of the search.

#### 3.1.4. Competition Rate and Support Rate

According to the introduction of the aforementioned interaction behaviors, the behavior of competing is a crucial interaction behavior. Therefore, in order to realistically simulate that behavior, this paper introduces the concept of the competition rate, which is used to control whether the competition terms are introduced when updating the candidates. Specifically, in each iteration, each candidate will introduce a competition term with a probability determined by the competition rate. This increases the randomness of the behavior of competing, making it more aligned with the behaviors in actual election processes. Below is the calculation formula for the competition rate and the judgment formula for introducing competitive elements:

(2) $$\begin{array}{l} R_{c}^{t}=R_{c,\min}+(R_{c,\max}-R_{c,\min})*\frac{cnt_{u,\max}-cnt_{u}^{t}}{cnt_{u,\max}}\\ f_{c}^{t}=\left\{\begin{array}{l} 1,\;\mathrm{rand}\leq R_{c}^{t}\\ 0,\;else \end{array}\right. \end{array}$$

where $R_{c}^{t}$ denotes the magnitude of the competition rate during the $t_{th}$ iteration. $R_{c,\min}$ and $R_{c,\max}$ denote the minimum and maximum competition rates. $cnt_{u}^{t}$ and $cnt_{u,\max}$ denote the number of iterations during which the leader remains unchanged at the $t_{th}$ iteration and the maximum number of iterations during which the leader remains unchanged. rand is random number uniformly distributed in the range of [0,1]. $f_{c}^{t}$ denotes whether the competition term is introduced during the $t_{th}$ iteration.

It can be seen from the above formula that the competition rate proposed in this paper is dynamically adjusted based on the operation of the algorithm. Specifically, the competition rate is inversely proportional to the number of iterations during which the leader remains unchanged. When the leader has not changed for a long time, the competition rate increases to promote the exploration behavior of the population. When the leader is frequently updated, the competition rate decreases to accelerate the convergence speed. Through this dynamically adjusted competition rate, it is possible to balance the exploration and exploitation behaviors of the population, thereby improving the search efficiency of the algorithm and the quality of the solutions. At the same time, the introduction of the competition rate allows the algorithm to adaptively adjust its search strategy to cope with complex optimization problems and dynamically changing search environments.

On the other hand, according to the introduction of the aforementioned behavior of supporting, an extremely important element is the degree of support that the voters and followers have for the candidates. In order to quantitatively describe this behavior of supporting of the voters and followers, this paper introduces the concept of the support rate, which ranges from 0 to 100, representing the degree of support that the voters and followers have for the candidates. Once the support rate is introduced, this behavior of supporting is simplified to the dynamic changes in the support rate of the voters and followers. Specifically, after each iteration, the voters update their support rates for the assigned candidates based on the changes in their own fitness values: if the change is positive, the support rate for the current candidate is increased; if the change is negative, the support rate is decreased. When the support rate for a certain candidate exceeds a predefined threshold (e.g., 80), this voter will become a follower of that candidate, with its support rate fixed at 100, while the support rates for other candidates drop to 0 and are no longer updated. And the above process of updating the support rate can be described using the following formula,

(3) $$\begin{array}{l} R_{s,i,m}^{t+1}=\left\{\begin{array}{l} R_{s,i,m}^{t}+R_{s,fixed},\;fit_{i}^{t+1}\geq fit_{i}^{t}\\ R_{s,i,m}^{t}-R_{s,fixed},\;else \end{array}\right.\\ R_{s,i,m}^{t+1}=\max(\min(R_{s,i,m}^{t+1},100),0) \end{array}$$

where $R_{s,i,m}^{t}$ denotes the support rate of the $i_{th}$ voter for the randomly assigned $m_{th}$ candidate during the $t_{th}$ iteration, ranging from 0 to 100. $R_{s,fixed}$ denotes the change in the support rate for each update. $\max\left(a,b\right)$ and $\min\left(a,b\right)$ return the larger and smaller of a and b.

#### 3.1.5. Learning Strategy

To introduce the electoral process into the standard PSO algorithm, thereby simulating the human interaction behaviors in the election processes, this paper makes targeted improvements to the learning strategies of particles at different levels based on the original standard PSO algorithm, as detailed below.

*The learning strategy of the leader*. As the optimal individual in the population, the leader’s position represents the current global best solution and is therefore usually not updated during each iteration.

*The learning strategy of the candidates*. As a group with the behavior of competing, the learning strategy of the candidates not only considers their own positions and the leader’s position but also includes a competition term to simulate individual differences in social competition. In summary, the following are the velocity and position update formulas for the candidates:

(4) $$\left\{\begin{array}{l} e_{c,i}^{t}=\frac{1}{N-1}\sum_{m=1}^{N}x_{m}^{t},(m\neq i)\\ v_{i}^{t+1}=\omega v_{i}^{t}+c_{1}r_{1}(pbest_{i}^{t}-x_{i}^{t})+c_{2}r_{2}(xleader^{t}-x_{i}^{t})-f_{c}^{t}c_{3}r_{3}(e_{c,i}^{t}-x_{i}^{t})\\ x_{i}^{t+1}=x_{i}^{t}+v_{i}^{t+1} \end{array}\right.$$

where t denotes the current iteration number. $e_{c,i}^{t}$ denotes the competition centroid of other candidates excluding the $i_{th}$ candidate during the $t_{th}$ iteration. N denotes the number of the candidates. $x_{i}^{t}$ and $v_{i}^{t}$ denote the position and velocity of the $i_{th}$ candidate during the $t_{th}$ iteration, respectively. w, $c_{1}$, $c_{2}$, and $c_{3}$ denote the inertia factor, individual cognitive learning factor, social cognitive learning factor, and competition factor, respectively, all of which are positive real numbers. $r_{1}$, $r_{2}$, and $r_{3}$ are random numbers uniformly distributed in the range of [0,1]. $pbest_{i}^{t}$ denotes the individual best position vector of the $i_{th}$ candidate during the $t_{th}$ iteration. $xleader^{t}$ denotes the position vector of the leader during the $t_{th}$ iteration. $f_{c}^{t}$ denotes the coefficient for introducing the competition term during the $t_{th}$ iteration.

*The learning strategy of the voters.* Since the voters are the key group for implementing the behavior of support in the algorithm, the learning strategy of the voters is extended from the standard PSO algorithm by adding a support term, so that the voters not only follow the leader but also perform personalized searches based on their own support rates for the candidates. In summary, the following are the velocity and position update formulas for the voters:

(5) $$\left\{\begin{array}{l} v_{i}^{t+1}=\omega v_{i}^{t}+c_{1}r_{1}(pbest_{i}^{t}-x_{i}^{t})+c_{2}r_{2}(xleader^{t}-x_{i}^{t})\\ \;+0.01*R_{s,i,m}^{t}c_{3}r_{3}(xcand_{m}^{t}-x_{i}^{t})\\ x_{i}^{t+1}=x_{i}^{t}+v_{i}^{t+1} \end{array}\right.$$

where $x_{i}^{t}$ and $v_{i}^{t}$ denote the position and velocity of the $i_{th}$ voter during the $t_{th}$ iteration, respectively. m is the index of the candidate randomly assigned to the voter during this iteration. $R_{s,i,m}^{t}$ denotes the support rate of the $i_{th}$ voter for the $m_{th}$ candidate during the $t_{th}$ iteration, ranging from 0 to 100. $c_{3}$ denotes the support factor. $r_{3}$ is a random number uniformly distributed in the range of [0,1]. $xcand_{m}^{t}$ denotes the position of the $m_{th}$ candidate randomly assigned.

*The learning strategy of the followers*. The followers are individuals among the voters whose support rate for a certain candidate exceeds a predefined threshold, making them a special group in the algorithm for implementing the behavior of supporting. Their learning strategy is similar to that of the voters, but since their support rate is fixed, it is no longer affected by the changes in the support rate. In summary, the following are the velocity and position update formulas for the followers,

(6) $$\left\{\begin{array}{l} v_{i}^{t+1}=\left\{\begin{array}{l} \omega v_{i}^{t}+c_{1}r_{1}(pbest_{i}^{t}-x_{i}^{t})+c_{2}r_{2}(xleader^{t}-x_{i}^{t})\\ \;+c_{3}r_{3}(xcand_{m}^{t}-x_{i}^{t}),\;m=k\\ \omega v_{i}^{t}+c_{1}r_{1}(pbest_{i}^{t}-x_{i}^{t})+c_{2}r_{2}(xleader^{t}-x_{i}^{t}),m\neq k \end{array}\right.\\ x_{i}^{t+1}=x_{i}^{t}+v_{i}^{t+1} \end{array}\right.$$

where $x_{i}^{t}$ and $v_{i}^{t}$ denote the position and velocity of the $i_{th}$ follower during the $t_{th}$ iteration, respectively. k denotes the index of the candidate that the current follower is following, so m=k indicates that the randomly assigned candidate is the one being followed, with the support rate of 100. Otherwise, the support rate is 0.

#### 3.1.6. Pseudocode of the Electoral Process

The pseudocode of the electoral process is shown in Algorithm 1.
**Algorithm 1:** The electoral process1:**For** each voter or follower2:        Update the voter or follower according to Equation (5) or Equation (6).3:        Calculate the individual fitness with the custom cost function and update the individual pbest.4:        Update the support rate according to Equation (3).5:        **If** the support rate of the voter is over fixed value, **then** change this voter to follower.6:**End**7:Calculate the competition rate according to Equation (2).8:**For** each candidate9:        **If** random value <= competition rate10:                Update the candidate with competition according to Equation (4).11:       
**Else**
12:                Update the candidate without competition according to Equation (4).13:       
**End**
14:        Calculate the individual fitness with the custom cost function and update the individual pbest.15:**End**16:Reselect the leader and candidates.17:**If** any candidate changes, **then** change its followers to voters.

### 3.2. Mutation Mechanism

#### 3.2.1. Introduction of Mutation Mechanism

The PSO algorithm with the introduced election mechanism not only effectively preserves suboptimal solutions but also significantly improves the diversity of the population, enabling a broader search for solutions in the early and middle stages of optimization. However, upon further consideration, the voters gradually converge as the optimization process progresses, and since candidates are elected from voter particles, this may inevitably cause the optimal and suboptimal solutions in the population to gradually converge in the later stages of optimization, potentially leading to being trapped in a local optimum.

Therefore, this paper further introduces a mutation mechanism based on the previous improvements, inspired by the genetic mutation process in biological evolution. This improvement aims to enhance population diversity and further improve algorithm accuracy by embedding the mutation mechanism in the update processes of the voters and followers, directly mutating the positions of particles.

#### 3.2.2. Mutation Rate

Although the introduction of the mutation mechanism significantly enhances population diversity and thereby improves algorithm accuracy, it also increases the proportion of exploration during the optimization process, resulting in a slower convergence speed. Therefore, when applying the mutation mechanism, it is essential to consider how to balance the accuracy and speed of the algorithm, which essentially involves designing the degree of mutation in the population during the optimization process.

To quantitatively describe and design the degree of mutation, this paper introduces the concept of a mutation rate, which represents the probability of particles undergoing mutation during the optimization process. Considering that the demand for solution exploration differs between the early and later stages of the optimization process, a method for dynamically adjusting the mutation rate is designed. Specifically, the mutation rate decreases as the number of iterations increases: the smaller the iteration number, the larger the mutation rate, and vice versa. The specific formula for dynamic adjustment of the mutation rate is

(7) $$R_{m}^{t}=R_{m,\min}+(R_{m,\max}-R_{m,\min})*\frac{{\left(iterations-i\right)}^{N_{m}}}{iterations^{N_{m}}}$$

where $R_{m}^{t}$ denotes the mutation rate at the $t_{th}$ iteration. $R_{m,\min}$ and $R_{m,\max}$ denote the minimum and maximum mutation rates during the optimization process, respectively. i and iterations denote the current iteration number and the maximum number of iterations. $N_{m}$ is a positive integer representing the exponent, typically set between 3 and 5.

According to the above formula, the proposed mutation rate is inversely proportional to the $N_{m}th$ power of the iteration count. This means that in the early stages of the optimization process, particles are more likely to undergo mutation, thereby increasing the algorithm’s exploratory capability. In the later stages, the mutation rate gradually decreases, and the algorithm transitions to fine-tuning and exploiting existing solutions, thus improving the convergence speed. This dynamic adjustment of the mutation rate helps accelerate its convergence while maintaining the algorithm’s exploratory capability, achieving a balance between algorithm accuracy and speed.

#### 3.2.3. Dynamic Mutation Strategy

For the mutation strategy, the simplest single mutation strategy is considered first. However, this paper argues that a single mutation method is not conducive to solving complex and variable optimization problems. Therefore, a dynamic mutation strategy is proposed. This strategy selects different mutation methods according to specific rules during each mutation in the optimization process, thereby enhancing the algorithm’s adaptability.

*The first mutation strategy: Random Mutation*. This mutation strategy is triggered with a relatively small probability. Its core idea is to completely randomize the particle’s position, akin to a “reinitialization” in the search space. In this study, it was found that this strategy is suitable for situations where the gaps between local optima are relatively large, helping particles escape the current region and explore new search spaces. However, it is more likely to affect the convergence speed. Specifically, after mutation, the particle’s position is set to a random location between the boundary’s minimum and maximum values, mathematically expressed as follows:

(8) $$x_{i}^{t+1}=b_{\min}+rand*(b_{\max}-b_{\min})$$

where $b_{\min}$ and $b_{\max}$ denotes the boundary’s minimum and maximum values, while rand is a random number uniformly distributed in the range of [0,1].

*The second mutation strategy: Cauchy Mutation*. This mutation strategy is adopted with a relatively high probability and is characterized by introducing the Cauchy distribution to increase the population diversity. In this study, it was found that this strategy is suitable for situations where the gaps between local optima are small, facilitating more detailed searches within local regions because of the limited jump range. Specifically, the position of the particle after mutation is generated by perturbing the current position using Cauchy mutation. The mathematical expression is as follows:

(9) $$x_{i}^{t+1}=x_{i}^{t}[1+\gamma \tan(\pi *(rand-0.5))]$$

where γ denotes the scaling parameter in the formula for the Cauchy distribution to control the mutation strength, while *rand* is a random number uniformly distributed in the range of [0,1].

Dynamic Selection of Mutation Strategies. In summary, the two mutation strategies mentioned above each have their respective advantages and disadvantages. Therefore, this paper adopts the following selection strategy for real-time decision-making:

(10) $$\mathrm{Mutation}\;\mathrm{Strategy}=\left\{\begin{array}{l} Random\;Mutation,\;rand\leq R_{m,fixed}\\ Cauchy\;Mutation,else \end{array}\right.$$

where *rand* is a random number uniformly distributed in the range of [0,1], while $R_{m,fixed}$ is a predefined constant, generally less than 0.5. According to this selection strategy, a fixed probability is used to choose different mutation strategies. Without significantly affecting computational complexity, this approach ensures a high probability of Cauchy mutation to enhance the local search capability, a low probability of random mutation to increase population diversity, and avoidance of the limitations associated with relying solely on a single mutation strategy.

#### 3.2.4. Pseudocode of Mutation Mechanism

The pseudocode of the mutation mechanism is shown in Algorithm 2.
**Algorithm 2:** The mutation mechanism1:Calculate the mutation rate according to Equation (7).2:**For** each voter or follower3:        **If** random value <= mutation rate4:                **If** random value <= fixed rate5:                        Update the voter or follower with random mutation according to Equation (8).6:                
**Else**
7:                        Update the voter or follower with Cauchy mutation according to Equation (9).8:                
**End**
9:        
**End**
10:**End**

### 3.3. Pseudocode and Flowchart of the IPSO Algorithm

**Figure 2:**
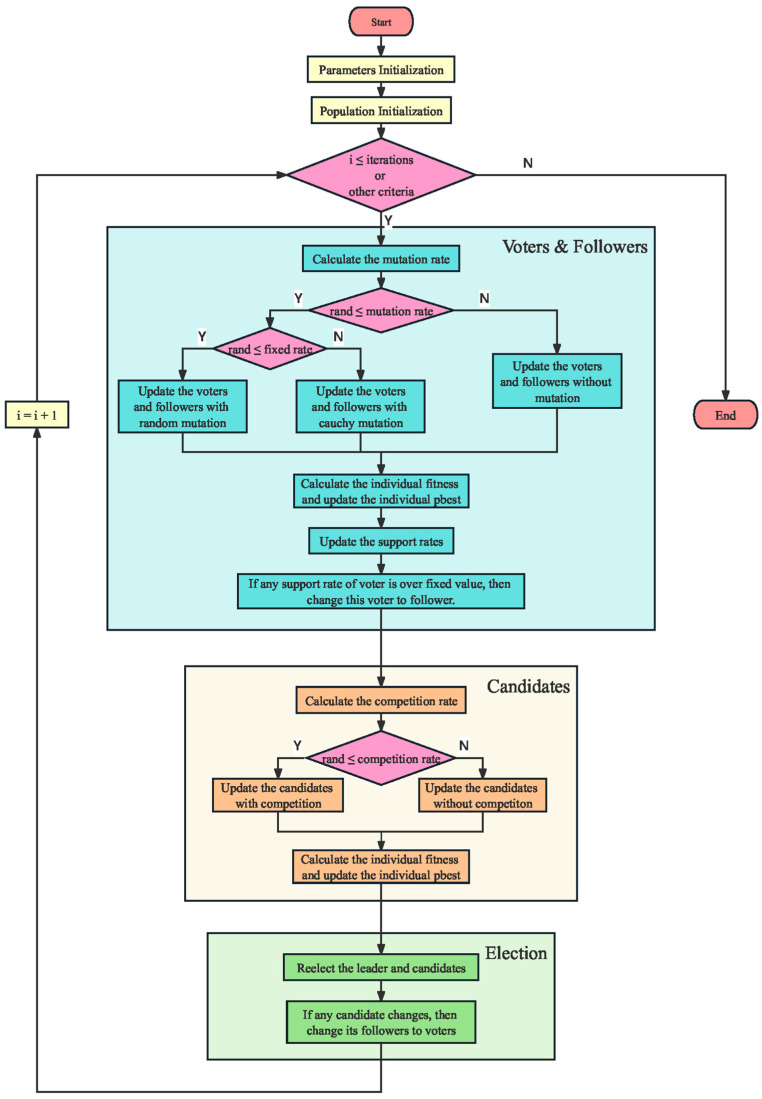
The flowchart of the IPSO algorithm.

In summary, IPSO is proposed in this paper to enhance the global search capability in the early and middle stages, while increasing population diversity in the middle and later stages. This leads to improved algorithm accuracy, thereby enhancing optimization performance. The pseudocode of the IPSO algorithm is shown in Algorithm 3, and the flowchart of this algorithm is shown in Figure 2.
**Algorithm 3:** The IPSO algorithm1:Set various parameters of the IPSO.2:Initialize the positions and velocities of population randomly, and calculate the fitness value.3:**Enter** main loop until the end condition is triggered.4:        Calculate the mutation rate according to Equation (7).5:        **For** each voter or follower6:                **If** random value <= mutation rate7:                        **If** random value <= fixed rate8:                                Update the voter or follower with random mutation according to Equation (8).9:                        
**Else**
10:                                Update the voter or follower with Cauchy mutation according to Equation (9).11:                        
**End**
12:                
**Else**
13:                        Update the voter or follower without mutation according to Equation (5) or Equation (6).14:                
**End**
15:                 Calculate the individual fitness with the custom cost function and up- date the individual pbest.16:                 Update the support rate according to Equation (3).17:                 **If** the support rate of the voter is over fixed value, **then** change this voter to follower.18:        
**End**
19:         Calculate the competition rate according to Equation (2).20:         **For** each candidate21:                **If** random value <= competition rate22:                        Update the candidate with competition according to Equation (4).23:                
**Else**
24:                        Update the candidate without competition according to Equation (4).25:                
**End**
26:                 Calculate the individual fitness with the custom cost function and up- date the individual pbest.27:       
**End**
28:        Reselect the leader and candidates.29:        **If** any candidate changes, **then** change its followers to voters.30:        i = i + 1.31:**Exit** main loop to end the optimization process.

## 4. Simulation Test and Result Analysis

To verify the specific performance of IPSO, this paper compares the algorithm with Particle Swarm Optimization (PSO), the Grey Wolf Optimizer (GWO), and the genetic algorithm (GA). The experimental environment is configured as follows: The operating system is Windows 11 (64-bit), the processor is an 11th Gen Intel(R) Core(TM) i7-11800H with a base frequency of 2.30 GHz, the memory is 16 GB, and the simulation software is MATLAB R2024b.

### 4.1. Comparison Algorithms and Parameter Settings

In this study, to fairly and comprehensively evaluate the performance of IPSO, we used the CEC2005 benchmark suite [35], which includes 25 test functions. These consist of five Unimodal Functions (F1–F5), seven Basic Multimodal Functions (F6–F12), two Expanded Functions (F13–F14), and eleven Composition Functions (F15–F25).

At the same time, this paper compares IPSO with three other classic swarm intelligence algorithms: PSO, GWO, and GA. To ensure the reliability of the test results, the population size for all algorithms was set to 55, and the number of iterations was set to 1000. The specific parameter settings for each algorithm are shown in Table 1.

### 4.2. Optimization Results and Analysis of Cec2005 Benchmark Functions

Using the aforementioned experimental environment and algorithm parameter settings, each algorithm was run independently 30 times on the 25 test functions. The results are shown in Table 2, which include metrics such as the average value, standard deviation, and best value. To more fairly evaluate the algorithm’s performance, this paper primarily uses the average of the experimental results to measure the optimization accuracy of the algorithms, and finally summarizes the experimental results of the 25 test functions to statistically analyze the optimization adaptability of the algorithms for different test functions.

When comparing the average values of the experimental results, it is evident that the IPSO algorithm provides significantly better solutions on most of the test functions, except for a few Unimodal Functions, demonstrating its strong global search capability. Additionally, the solutions obtained by the IPSO algorithm on certain Unimodal Functions are only slightly inferior to those of the standard PSO algorithm, showing its outstanding local search ability. Furthermore, comparing the standard deviations of the experimental results clearly shows that the IPSO algorithm has higher stability compared to the other algorithms, highlighting its superior adaptability.

In conclusion, IPSO retains the excellent local search capability of the original algorithm (PSO) while significantly enhancing its global search ability, resulting in an improved optimization accuracy and a noticeable increase in the algorithm’s adaptability to different optimization problems.

### 4.3. Convergence Curve Analysis

**Figure 3:**
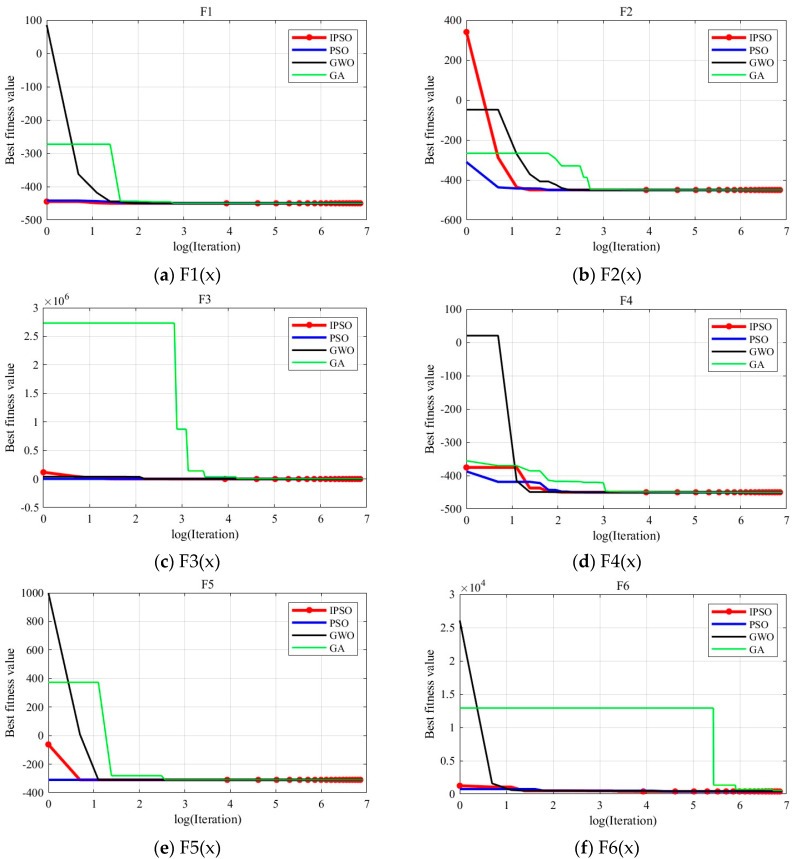

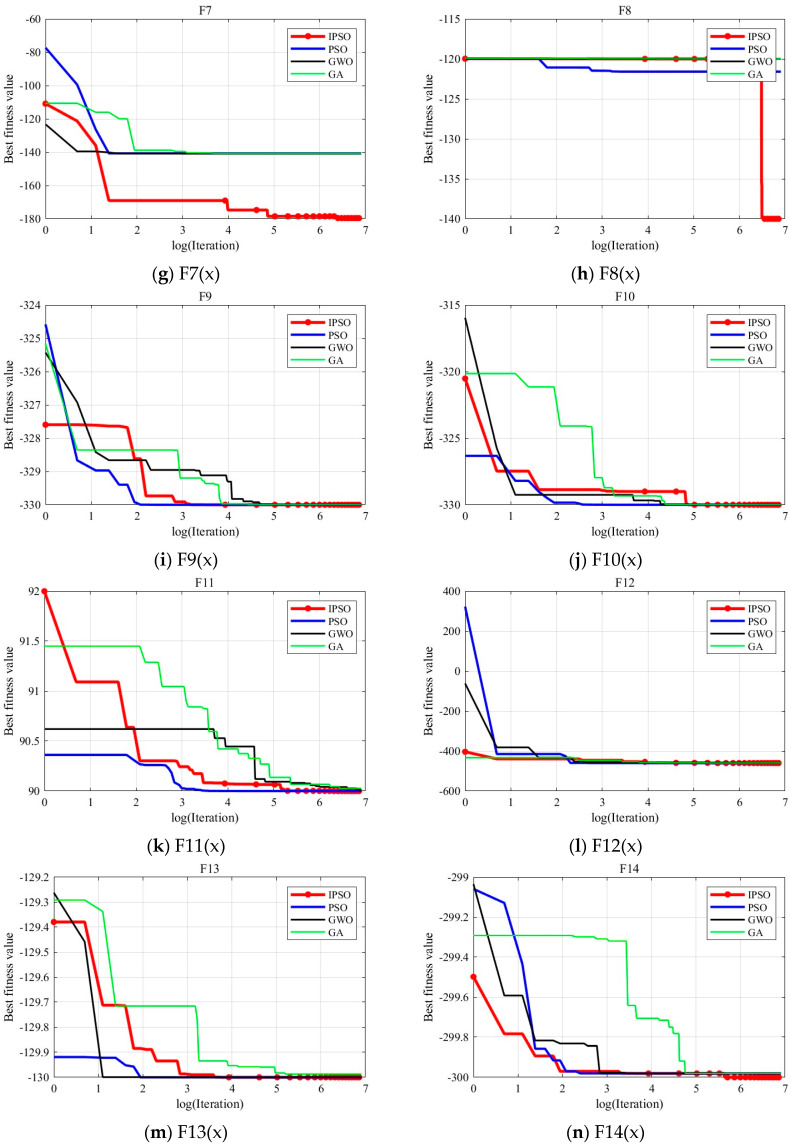

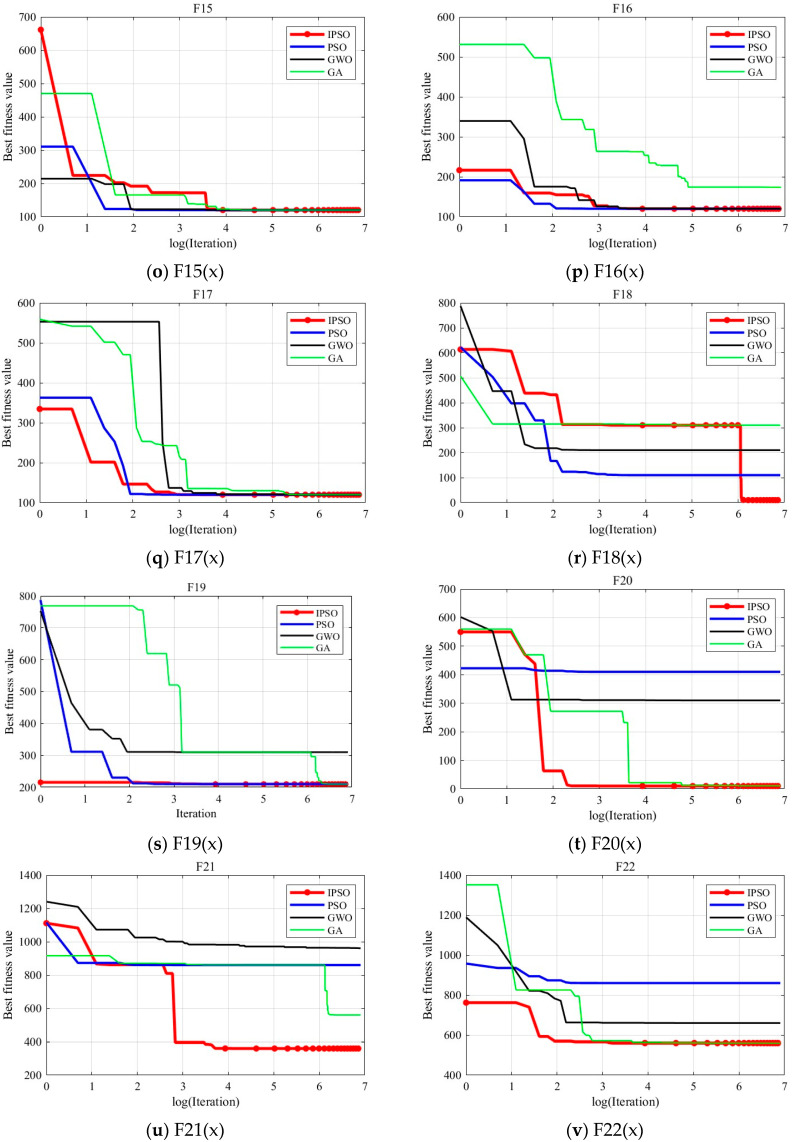

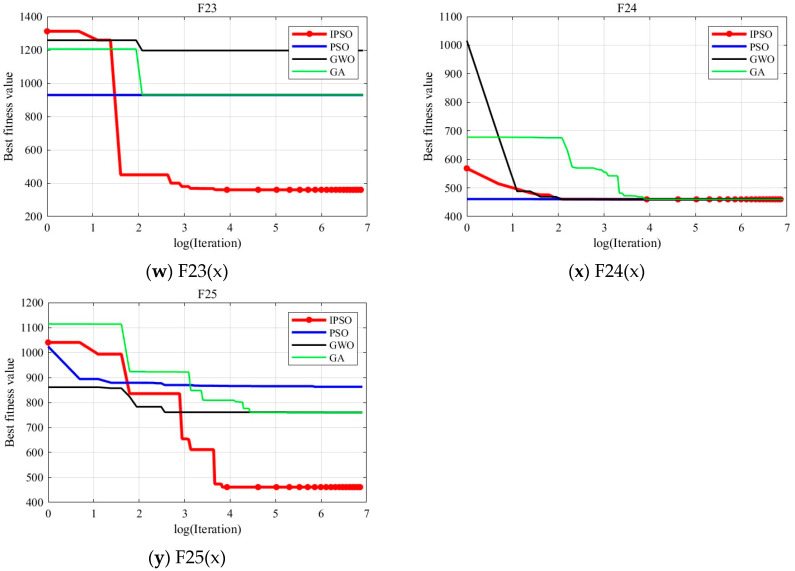
Convergence curves of test functions.

To visually illustrate the optimization accuracy and convergence speed of the algorithm, this paper analyzes the results using convergence curves. Figure 3 shows the convergence curves obtained from the 15th run of the experiment. The red, blue, black, and green curves represent the convergence of the IPSO, PSO, GWO, and GA algorithms, respectively. The *x*-axis of each curve represents the logarithm of the number of iterations, and the *y*-axis represents the fitness value of the optimal solution during the optimization process.

From the Figure 3, it can be inferred that IPSO’s optimization accuracy generally outperforms the PSO, GWO, and GA algorithms. For some Multimodal Functions and Composition Functions where other algorithms perform poorly, IPSO continues to optimize the current global optimal solution in the later stages of the optimization process, although slightly slower in convergence speed. While other algorithms fall into local optima, IPSO still achieves better results, demonstrating its advantage in solving complex optimization problems.

In summary, within the specified number of iterations, IPSO shows a significant advantage in optimization accuracy.

## 5. Application of IPSO in Urban Drone Path Planning

### 5.1. Environmental Modeling

For the 3D urban path-planning problem, the modeling of the urban environment is one of the key aspects. In this study, a three-dimensional space of 100 m×100 m×100 m is selected as the flight area for the drone, with obstacles of different shapes simulating real-world environments such as city buildings. To facilitate the modeling, cuboids and cylinders are used to build the obstacles. The positions of the cuboids are determined by their upper and lower vertices. The cylinders are determined using the center coordinates, height, and radius. The specific obstacle location information is shown in Table 3. All simulation experiments are conducted in this environment.

### 5.2. Cost Function

The cost function is used to evaluate the quality of the planned path. The smaller the value of the cost function, the better the quality of the obtained path. Similar to general path-planning problems, the cost function for urban drone path planning consists of a series of different cost components, such as path length cost, height cost, collision cost, and path smoothness cost. For simplicity, this study considers only the path length, height, and collision costs.

#### 5.2.1. Path Length Cost

Path length is the most direct method to evaluate the quality of a planned path. Since the drone has limited endurance, the shorter the planned path, the more advantageous it is for the drone’s mission. In this study, the path is composed of a predefined start point, predefined end point, and $N_{p}$ path points to be optimized along the path. The coordinates of the $j_{th}$ path point on the $i_{th}$ path are denoted as $X_{ij}=(x_{ij},y_{ij},z_{ij})$. Therefore, the path length cost is defined as the sum of the Euclidean distances between adjacent path points from the start to the end, as expressed by the following equation,

(11) $$cost_{i,length}=\sum_{j=0}^{N_{p}}\sqrt{{\left(x_{i(j+1)}-x_{ij}\right)}^{2}+{\left(y_{i(j+1)}-y_{ij}\right)}^{2}+{\left(z_{i(j+1)}-z_{ij}\right)}^{2}}$$

where $\left(x_{i0},y_{i0},z_{i0}\right)$ denotes the coordinates of the path start point, and $\left(x_{i(N_{p}+1)},y_{i(N_{p}+1)},z_{i(N_{p}+1)}\right)$ denotes the coordinates of the path end point.

#### 5.2.2. Height Cost

Choosing the correct flight height is also crucial for drone path planning. In drone path planning, especially in urban path planning, the setting of flight height is generally mandatory, as both excessively low and high flight heights may lead to accidents. Therefore, the cost function for flight height is expressed as follows,

(12) $$\begin{array}{l} isoverheight(X_{ij})=\left\{\begin{array}{l} \infty ,\;unsafe\\ 0\;,\;safe \end{array}\right.\\ cost_{i,height}=\sum_{j=1}^{N_{p}}isoverheight(X_{ij}) \end{array}$$

where isoverheight( ) is a custom function used to check whether the height of the path point is within the prescribed range. If the height exceeds the limit, the cost is set to infinite; if the height is within the normal range, the cost is set to zero.

#### 5.2.3. Collison Cost

Collision-free is the most important criterion in path planning. In urban drone path planning, the safety of the drone’s flight is the premise of the planning. Therefore, once a collision occurs, the cost function value should make the path invalid, meaning the path’s cost should be infinitely large. Thus, the cost function for collision is expressed as follows:

(13) $$\begin{array}{l} iscollison(X_{ij},X_{i(j+1)})=\left\{\begin{array}{l} \infty ,\;unsafe\\ 0,\;safe \end{array}\right.\\ cost_{i,collison}=\sum_{j=0}^{N_{p}}iscollison(X_{ij},X_{i(j+1)}) \end{array}$$

where iscollison( ) is a custom function used to determine whether the path between two adjacent points will collide with obstacles. If a collision occurs, the cost is set to infinity; if no collision occurs, the cost is set to zero. In this study, it is assumed that a collision occurs if the path points overlap with the obstacle locations in the inflated 3D grid map.

#### 5.2.4. Total Cost Function

The total cost function is defined as the combination of the three individual cost functions mentioned above:

(14) $$cost_{i}=cost_{i,length}+cost_{i,height}+cost_{i,collison}$$

### 5.3. Path Segmentation Optimal Update Algorithm

In drone path-planning tasks, planning efficiency is often crucial. A faster path-planning process can greatly improve task execution efficiency, thus increasing the overall benefits. However, it has been found in the research that, during path planning using IPSO, the convergence speed tends to slow down significantly in the later stages of the optimization process, leading to a decrease in planning speed. This phenomenon occurs because the path oscillates around the optimal path solution, and the more path points there are, the more intense the oscillation becomes, thereby greatly reducing the convergence speed of path planning. Following analysis, it is proposed that the cause of this phenomenon lies in the fact that each path is composed of multiple path points. Therefore, during particle updates, multiple path points are updated simultaneously. The path points with bad positions may cause updates to the path points with good positions, leading to the movement of original optimal path points to suboptimal positions due to factors like inertia. This oscillation, when repeated, results in the phenomenon observed in the experiment.

To reduce or even avoid the aforementioned oscillation phenomenon, this paper proposes a path segmentation optimal update algorithm. The specific idea is as follows: In the later stages of the optimization process, during each iteration, the path containing N path points is decomposed into N updates. Each update only updates one path point, which is randomly selected from the un-updated path points. After each update, the fitness value of the path is calculated using Equation (17), and the optimal path from these N updates is selected as the new path for this iteration. This algorithm refines the original path update process by updating path points step by step instead of updating the entire path at once. This approach reduces or even eliminates the oscillation phenomenon, significantly improving the efficiency and convergence speed of path planning.

(15) $$fit_{i}^{t+1}=fit_{i}^{t}-fit_{ij}^{t}+fit_{ij}^{t+1}$$

where $fit_{i}^{t+1}$ and $fit_{i}^{t}$ denote the fitness values of the $i_{th}$ path after and before the update, respectively. $fit_{ij}^{t+1}$ and $fit_{ij}^{t}$ denote the fitness values of the updated and original path points j relative to the previous and next path points.

### 5.4. Path Smoothing

The improved PSO algorithm proposed in this study defines each path as consisting of a starting point, an end point, and several intermediate path points. The connections between these points are represented as straight line segments, which inevitably result in numerous sharp turns. When drones follow such paths during flight, excessive turns can significantly increase energy consumption. Therefore, it is essential to optimize the sharp turns on the path, a process referred to as path smoothing.

In comparison with a series of path-smoothing algorithms, this study selected the cubic B-spline algorithm due to its advantages in local control, flexibility, and computational efficiency. The smoothed path is expressed as follows:

(16) $$C(t)=\sum_{i=0}^{n}P_{i}B_{i,3}(t)$$

where C(t) denotes the path expression. n is the number of control points. $P_{i}$ denotes the $i_{th}$ control point. $B_{i,3}(t)$ denotes is the $i_{th}$ cubic B-spline basis function, which is recursively defined as follows:

(17) $$\left\{\begin{array}{l} B_{i,0}(z)=\left\{\begin{array}{l} 1,{\;z}_{i}\leq z\leq z_{i+1}\\ 0,\;\mathrm{else} \end{array}\right.\\ B_{i,k}(z)=\frac{z-z_{i}}{z_{i+k}-z_{i}}B_{i,k-1}(z)+\frac{z_{i+k+1}-z}{z_{i+k+1}-z_{i+1}}B_{i+1,k-1}(z) \end{array}\right.$$

where $B_{i,k}(t)$ denotes the $i_{th}$ B-spline basis function of $k_{th}$ order. $z_{i}$ denotes the $i_{th}$ knot, which defines the segmentation of the curve.

### 5.5. Urban Drone Path Planning Simulation

**Figure 4:**
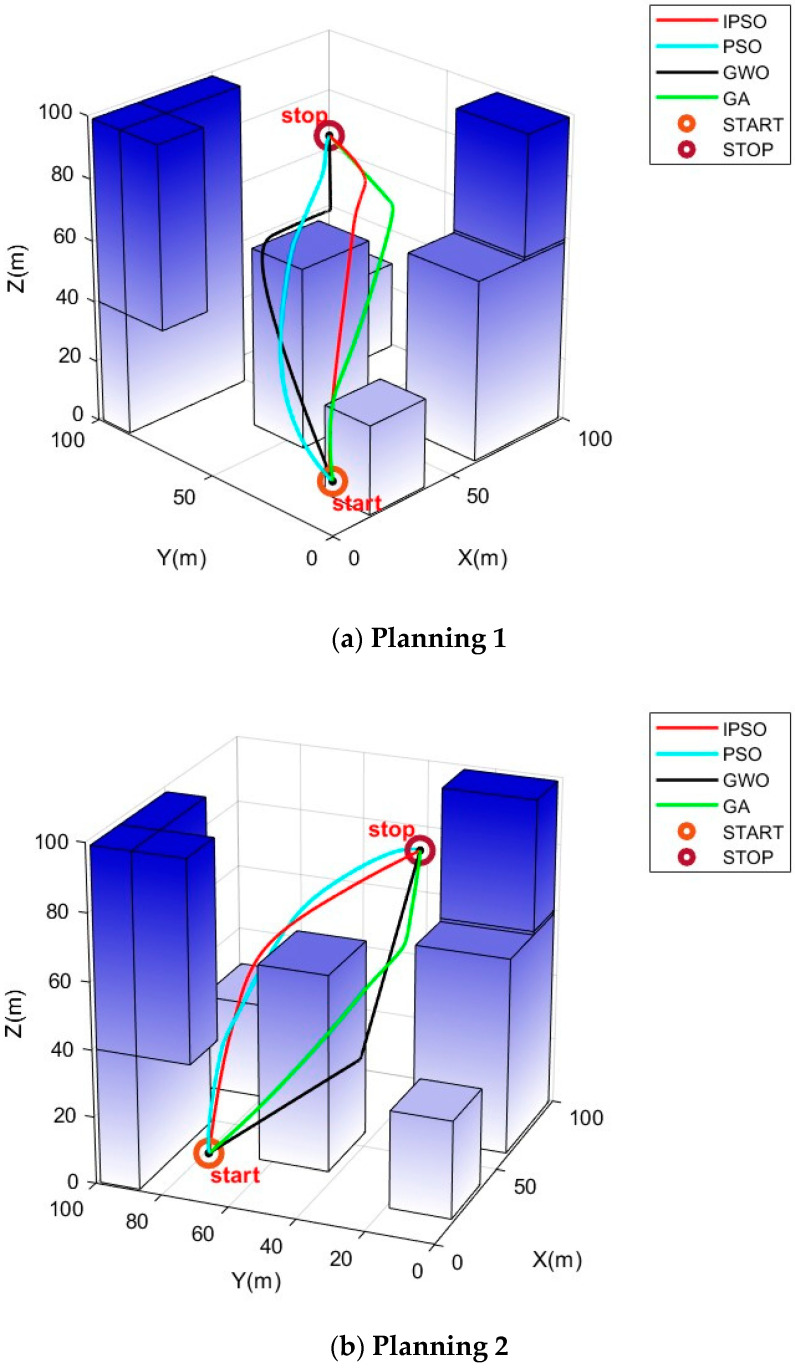

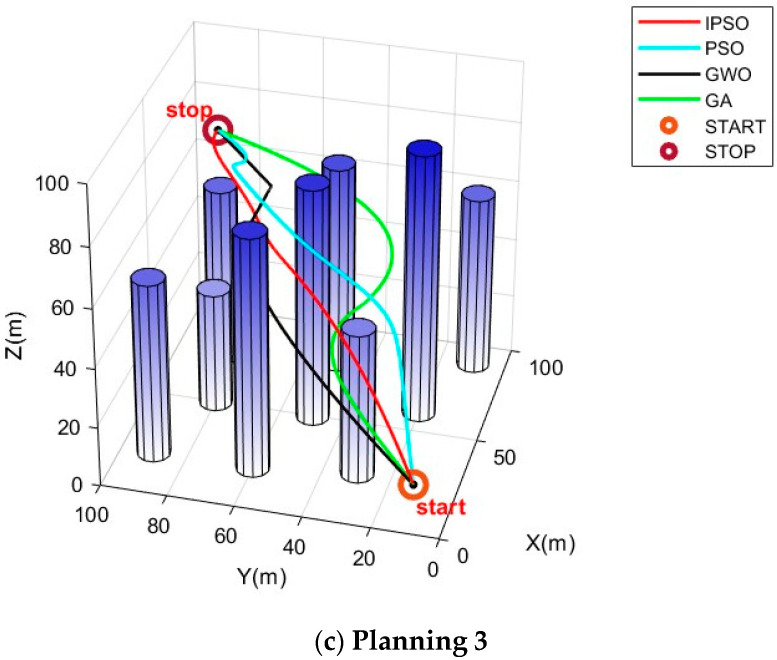
Urban drone path planning.

**Figure 5:**
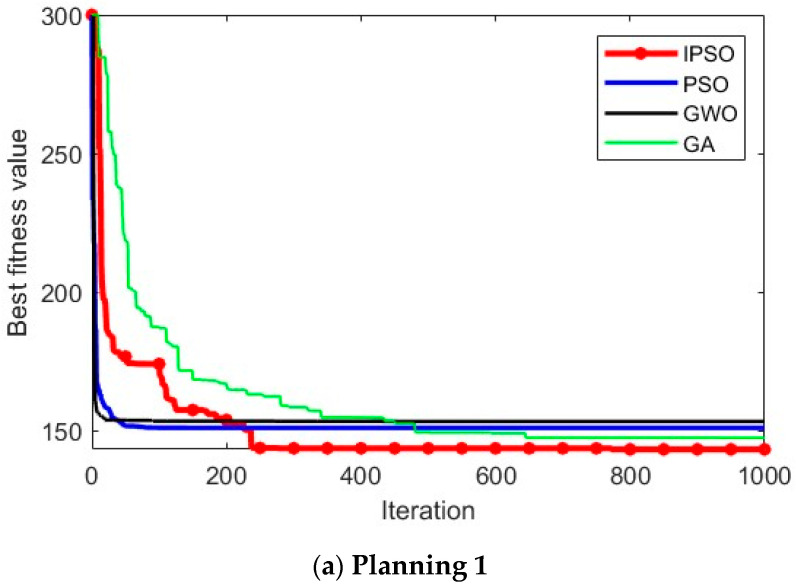

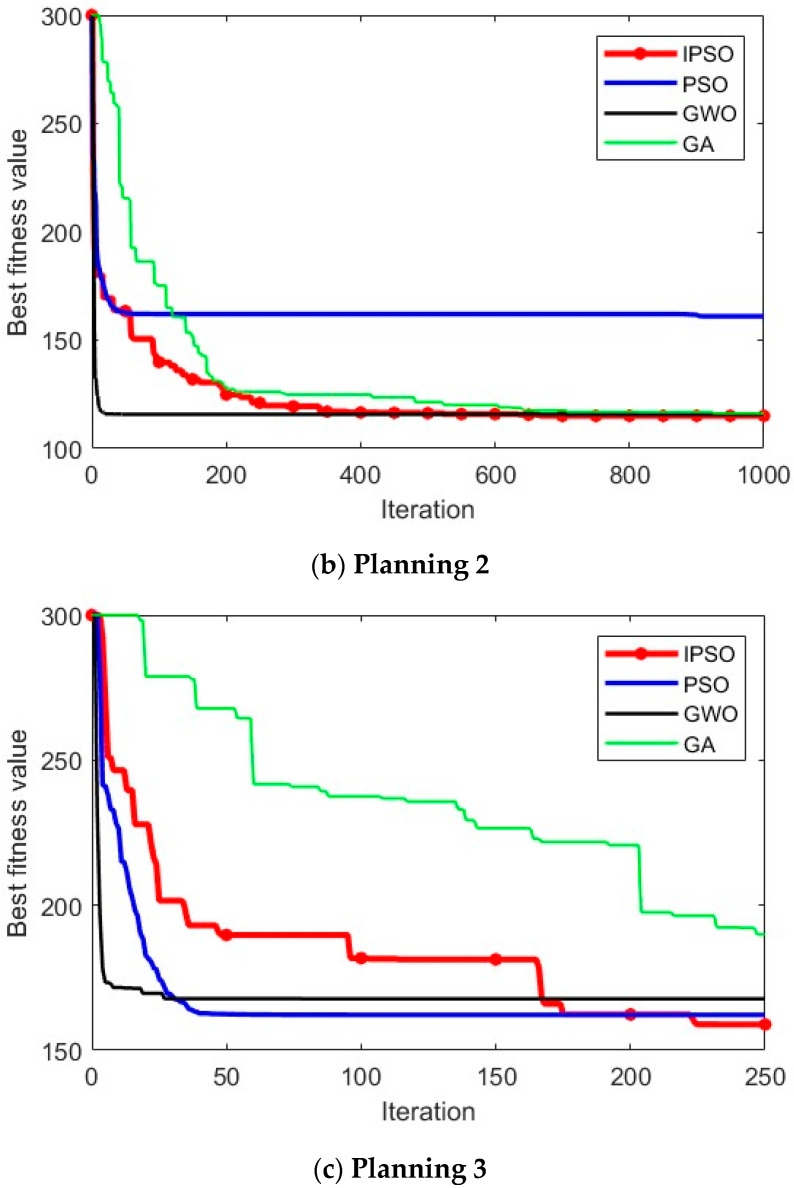
Convergence curve of urban drone path planning.

In this study, path-planning simulation experiments were conducted within the urban environment model described in the Section 5.1 on environment modeling. We considered different scenarios and different start and end points to test the optimization ability of the algorithm. Similar to the earlier benchmark experiments, the IPSO algorithm was compared with three other swarm intelligence optimization algorithms, namely PSO, GWO, and GA, in these path-planning simulations. Each algorithm was independently executed 20 times in the environment for comparison and analysis. To ensure the reliability of the results, the population size for all algorithms was set to 55, with 1000 iterations, and the other parameters for the algorithms remained consistent with those in Table 1. Specifically, each path consisted of six intermediate path points. The simulation results are shown in Figure 4 and Figure 5. In (a) and (c), the start point is (10, 10, 10) and the end point is (90, 90, 70). In (b), the start point is (10, 70, 10) and the end point is (80, 35, 80).

In Figure 4, it can be visually observed that, in the situations of different scenarios and different start and end points, IPSO outperforms the other three algorithms in planning superior paths, demonstrating its excellent optimization capability and robust adaptability. Figure 5 provides more detailed insights, showing that the paths planned by the IPSO algorithm are consistently shorter than those generated by the other three algorithms. This reduction in path length leads to shorter flight times and significantly enhances task execution efficiency, indicating a superior path-planning performance.

Table 4 presents the results of path planning, including metrics such as the average value, standard deviation, best value, and worst value.

When comparing the average values of the experimental results, it is evident that the cost of the path solutions obtained by the IPSO algorithm is significantly lower than that of the other algorithms, indicating that its path solutions are markedly superior. This demonstrates that the improved algorithm effectively enhances the search capability and algorithm accuracy. Furthermore, when analyzing the standard deviation of the experimental results, it can be found that IPSO consistently delivers stable and superior optimization results for path-planning problems under different starting points, highlighting its strong adaptability. In summary, the IPSO algorithm exhibits an outstanding performance in addressing urban drone path-planning problems.

**Figure 6:**
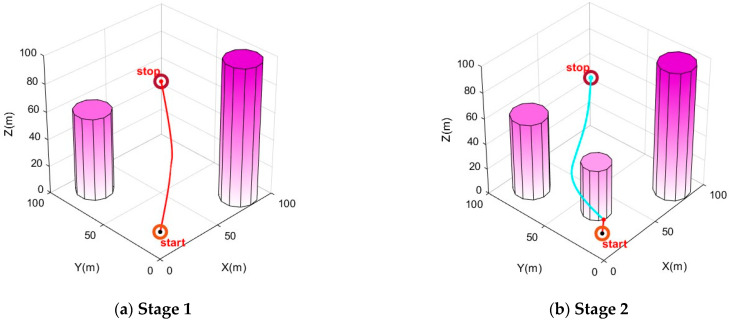

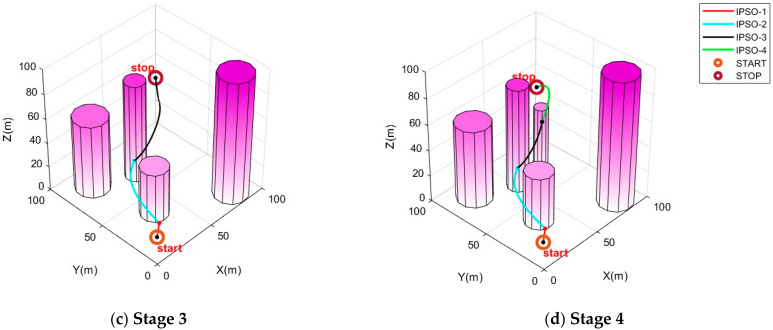
IPSO-based dynamic replanning strategy.

Furthermore, to address safety-critical challenges posed by environmental uncertainties or sensor noise in drone path planning, this research proposes an IPSO-based dynamic replanning strategy. The urban environment is modeled with latent obstacle estimation, and it is assumed that the drone will hover or execute a controlled landing during path recalculation to ensure operational continuity. As illustrated in Figure 6, the experimental stages are as follows. In Stage 1, the cylindrical obstacles represent the original grid map modeled before takeoff, while the red path denotes the path planned based on the initial map. When the drone follows the red path and reaches a certain distance, new cylindrical obstacles (shown in Stage 2) are dynamically introduced to simulate newly detected obstacles during flight. At this point, the IPSO algorithm reinitializes path planning from the drone’s current position (retaining the original end point), generating the blue path based on the updated grid map. Additional obstacles (shown in Stages 3 and 4) are successively introduced during flight, triggering further replanning to generate the black and green paths, respectively. Experimental results demonstrate that the IPSO algorithm with the dynamic replanning strategy can effectively avoid sudden obstacles not present in the original grid map. This provides an effective solution for handling environmental uncertainties or sensor noise in IPSO-based path planning.

## 6. Conclusions

This paper addresses the urban drone path-planning problem by proposing an IPSO algorithm. By incorporating swarm intelligence and the competitive and collaborative behaviors observed in human electoral process, the global search capability and optimization accuracy of IPSO are significantly enhanced. The utilization of hierarchical structures, competitive mechanisms, and dynamic mutation strategies effectively overcomes the standard PSO’s tendency to fall into local optima when dealing with complex optimization problems. Experimental results using the CEC2005 benchmark test functions demonstrate that the IPSO algorithm outperforms the standard PSO, GWO, and GA in optimizing multiple test functions. Particularly in the optimization of Multimodal and Composition Functions, IPSO shows a stronger ability to continuously optimize the global optimum, indicating a superior performance in solving complex problems.

In the practical application of urban UAV path planning, this paper further introduces a path segmentation optimal update algorithm and a cubic B-spline algorithm based on the IPSO algorithm. The proposed algorithm significantly reducing path length and energy consumption, improving the smoothness and planning efficiency of drone paths while enhancing flight safety in complex urban environments. Simulation experiments verify that the proposed path-planning algorithm exhibits an excellent performance across various test scenarios with different starting and ending points, generating shorter paths and flight times with higher stability and adaptability. Future research directions will include further optimizing the algorithm’s dynamic adjustment mechanisms to improve convergence speed and adaptability, as well as incorporating more advantages from biological intelligence. However, the proposed IPSO algorithm still faces limitations in computational efficiency. This restricts its applicability in scenarios requiring high real-time performance. For application in urban drone path planning, IPSO is only used for global path planning generally. If there is a real-time requirement, it can be considered in combination with the local path-planning algorithm. We will also continue to optimize the algorithm by optimizing the dynamic adjustment mechanisms, introducing heuristic strategies, and so on in future research, to improve the operational efficiency of IPSO and further expand its application.

## Figures and Tables

**Table 1:** Algorithm parameter settings.

Algorithm	Main Parameters
IPSO	$N=5,\;w=0.7,\;c_{1}=1.5,\;c_{2}=1.5,\;c_{3}=1.5$ $R_{c,\max}=50,\;R_{c,\min}=10,\;cnt_{u,\max}=iterations/8,{\;R}_{s,fixed}=10$ $R_{m,\max}=30,\;R_{m,\min}=10,\;N_{m}=5,\;R_{m,fixed}=0.4$
PSO	$w=0.9,\;c_{1}=1.5,\;c_{2}=1.5$
GWO	-
GA	$R_{\mathrm{mutation}}=0.1,\;R_{cross}=0.5$

**Table 2:** Optimization results of cec2005 benchmark functions.

Test Function	Indicator	IPSO	PSO	GWO	GA
F1(x)	**Avg**	−450	−450	−449.999999	−425.952248
	**Std**	2.111112 × 10^−14^	0	1.820577 × 10^−6^	25.555973
	**Best**	−450	−450	−450	−449.534698
	**Ranking**	2	1	3	4
F2(x)	**Avg**	−450	−450	−449.999994	−405.596209
	**Std**	1.165899 × 10^−13^	0	8.731787 × 10^−6^	52.953349
	**Best**	−450	−450	−450	−449.447446
	**Ranking**	2	1	3	4
F3(x)	**Avg**	−450	−291.718656	1659.3677704	3277.750098
	**Std**	4.095846 × 10^−7^	866.942621	2376.386817	3528.763719
	**Best**	−450	−450	−449.961901	−401.367885
	**Ranking**	1	2	3	4
F4(x)	**Avg**	−450	−450	−449.999994	−416.461782
	**Std**	1.411290 × 10^−12^	0	7.065243 × 10^−6^	42.570312
	**Best**	−450	−450	−450	−446.094106
	**Ranking**	2	1	3	4
F5(x)	**Avg**	−310	−310	−310	−310
	**Std**	0	0	0	0
	**Best**	−310	−310	−310	−310
	**Ranking**	1	1	1	1
F6(x)	**Avg**	390	400.026165	454.317210	416.378099
	**Std**	1.125253 × 10^−11^	38.692063	40.952152	39.244906
	**Best**	390	390	390	390.002167
	**Ranking**	1	2	4	3
F7(x)	**Avg**	−178.307870	−140.506148	−140.836896	−137.325516
	**Std**	0.947948	0.453619	0.032987	7.992522
	**Best**	−179.288269	−140.721283	−140.847181	−140.173508
	**Ranking**	1	3	2	4
F8(x)	**Avg**	−140	−123.999641	−120.664410	−119.760441
	**Std**	0	8.136944	3.639764	0.259870
	**Best**	−140	−140	−139.935693	−120.536663
	**Ranking**	1	2	3	4
F9(x)	**Avg**	−330	−329.568851	−329.801007	−314.726714
	**Std**	0	0.501466	0.404787	6.0152092
	**Best**	−330	−330	−330	−325.883823
	**Ranking**	1	3	2	4
F10(x)	**Avg**	−330	−329.436189	−329.834087	−307.451342
	**Std**	1.055555 × 10^−14^	0.565455	0.743327	12.665757
	**Best**	−330	−330	−330	−325.769611
	**Ranking**	1	3	2	4
F11(x)	**Avg**	90.000020	90.008766	90.014066	90.123873
	**Std**	3.075193 × 10^−5^	0.047917	0.051661	0.081992
	**Best**	90	90	90.000906	90.027348
	**Ranking**	1	2	3	4
F12(x)	**Avg**	−460	−460	−459.652625	−392.819433
	**Std**	5.850990 × 10^−11^	0	0.443965	56.504576
	**Best**	−460	−460	−459.999986	−459.999489
	**Ranking**	2	1	3	4
F13(x)	**Avg**	−129.999342	−129.970727	−129.987677	−125.716794
	**Std**	0.003602	0.038282	0.015810	11.192063
	**Best**	−130	−130	−130	−129.937801
	**Ranking**	1	3	2	4
F14(x)	**Avg**	−299.992227	−299.979382	−299.984732	−299.030191
	**Std**	0.009682	0.010767	0.007020	0.057294
	**Best**	−300	−300	−300	−299.207456
	**Ranking**	1	3	2	4
F15(x)	**Avg**	120	173.438024	171.423962	133.593857
	**Std**	4.678729 × 10^−13^	52.155256	51.094450	44.552562
	**Best**	120	120	120	120
	**Ranking**	1	4	3	2
F16(x)	**Avg**	120	155.102276	172.327423	165.117313
	**Std**	1.371207 × 10^−14^	42.705134	102.110015	42.324586
	**Best**	120	120	120	120
	**Ranking**	1	2	4	3
F17(x)	**Avg**	120	143.661025	152.204302	160.281951
	**Std**	1.730435 × 10^−14^	32.299819	48.072136	37.710854
	**Best**	120	120	120	120
	**Ranking**	1	2	3	4
F18(x)	**Avg**	10	300	246.810454	266.812006
	**Std**	9.886142 × 10^−7^	118.467222	82.393323	127.507629
	**Best**	10	10	110.066944	10.258672
	**Ranking**	1	4	2	3
F19(x)	**Avg**	170.083801	340	269.280453	300.223773
	**Std**	56.232486	102.216808	71.880495	70.592002
	**Best**	10.018245	210	112.226819	113.127308
	**Ranking**	1	4	2	3
F20(x)	**Avg**	20	273.333333	254.623740	210.357130
	**Std**	30.512857	129.942516	89.219680	159.311326
	**Best**	10	10	111.721298	10.003517
	**Ranking**	1	4	3	2
F21(x)	**Avg**	366.666666	719.622594	661.015170	613.797966
	**Std**	36.514837	168.549209	204.535187	88.241399
	**Best**	360	360	360.000001	560.000024
	**Ranking**	1	4	3	2
F22(x)	**Avg**	446.666686	659.881575	648.032910	628.196021
	**Std**	100.801368	146.385566	60.846282	87.267175
	**Best**	360	360	495.632347	560.000140
	**Ranking**	1	4	3	2
F23(x)	**Avg**	385.389456	829.452956	683.332953	712.911694
	**Std**	77.470487	182.859350	339.324722	152.364963
	**Best**	360	360	360	360.001968
	**Ranking**	1	4	2	3
F24(x)	**Avg**	450	469.150180	456.260876	460.002078
	**Std**	30.512857	38.524190	18.565121	0.004279
	**Best**	360	460	358.508267	460.000003
	**Ranking**	1	4	2	3
F25(x)	**Avg**	437.625668	732.660497	785.231299	749.432197
	**Std**	43.573251	166.982200	207.169366	96.469473
	**Best**	360	360	360.054591	419.794733
	**Ranking**	1	2	4	3
**Average Ranking**		1.16	2.64	2.68	3.28
**Final Ranking**		1	2	3	4

**Table 3:** The obstacle location information.

Scenario	Architecture	Lower Vertex	Upper Vertex
Scenario 1	Building 1	(15, 0, 0)	(40, 20, 30)
	Building 2	(30, 42, 0)	(60, 65, 60)
	Building 3	(70, 70, 0)	(100, 100, 30)
	Building 4	(0, 86, 0)	(50, 100, 40)
	Building 5	(0, 86, 40)	(50, 100, 100)
		(0, 70, 40)	(20, 86, 100)
	Building 6	(60, 0, 0)	(100, 30, 60)
		(80, 0, 60)	(100, 30, 100)
Scenario 2	**Architecture**	Center X, Center Y, Height, Radius	
	Building 1	(25, 30, 50, 5)	
	Building 2	(75, 85, 60, 5)	
	Building 3	(60, 20, 90, 5)	
	Building 4	(80, 50, 70, 5)	
	Building 5	(50, 50, 80, 5)	
	Building 6	(20, 60, 80, 5)	
	Building 7	(50, 80, 40, 5)	
	Building 8	(90, 10, 60, 5)	
	Building 9	(20, 90, 60, 5)	

**Table 4:** Path planning results under two different starting points.

Test Function	Indicator	IPSO	PSO	GWO	GA
Planning 1	**Avg**	144.296760	166.284557	157.449830	151.865283
	**Std**	2.033130	22.186115	12.906774	8.138659
	**Best**	141.383928	147.386272	153.955209	141.692737
	**Worst**	148.170932	214.948553	204.104464	167.703344
	**Ranking**	1	4	3	2
Planning 2	**Avg**	113.776126	135.392295	114.903634	122.945473
	**Std**	1.002453	16.943708	0.706795	9.395027
	**Best**	111.782006	114.349097	114.346174	115.905400
	**Worst**	115.096053	164.877072	117.397439	146.093836
	**Ranking**	1	4	2	3
Planning 3	**Avg**	137.030056	153.006240	139.566073	173.011788
	**Std**	17.597429	21.340279	16.574379	17.939675
	**Best**	119.174606	125.422954	121.780195	131.703866
	**Worst**	171.895310	186.264765	170.414441	206.094407
	**Ranking**	1	3	2	4
**Average Ranking**		1	3.67	2.33	3
**Final Ranking**		1	4	2	3

## Data Availability

Data are contained within the article.
